# Misdiagnosis and Mistreatment of Post-Kala-Azar Dermal Leishmaniasis

**DOI:** 10.1155/2013/351579

**Published:** 2013-02-21

**Authors:** Ahmed Mohamed El Hassan, Eltahir Awad Gasim Khalil, Waleed Mohamed Elamin, Lamyaa Ahmed Mohamed El Hassan, Mogtaba Elsaman Ahmed, Ahmed Mudawi Musa

**Affiliations:** ^1^Leishmaniasis Research Group, Institute of Endemic Diseases, University of Khartoum, Medical Campus, Qasr Avenue, P.O. Box 102, 11111 Khartoum, Sudan; ^2^Department of Clinical Pathology and Immunology, Institute of Endemic Diseases, University of Khartoum, P.O. Box 45235, 11111 Khartoum, Sudan

## Abstract

Post-kala-azar dermal leishmaniasis (PKDL) is a known complication of visceral leishmaniasis (VL) caused by *L. donovani*. It is rare in VL caused by *L. infantum* and *L. chagasi*. In Sudan, it occurs with a frequency of 58% among successfully treated VL patients. In the majority of cases, PKDL can be diagnosed on the basis of clinical appearance, distribution of the lesions, and past history of treated VL. The ideal diagnostic method is to demonstrate the parasite in smears, by culture or PCR. Diagnosis is particularly difficult in patients who develop PKDL in the absence of previous history of visceral leishmaniasis. We describe a case of cutaneous leishmaniasis misdiagnosed as PKDL and 3 cases of PKDL who were either misdiagnosed or mistreated as other dermatoses. This caused exacerbation of their disease leading to high parasite loads in the lesions and dissemination to internal organs in one of the patients, who was also diabetic. The latter patient had *L. major* infection. A fourth patient with papulonodular lesions on the face and arms of 17-year duration and who was misdiagnosed as having PKDL is also described. He turned out to have cutaneous leishmaniasis due to *L. major*. Fortunately, he was not treated with steroids. He was cured with intravenous sodium stibogluconate.

## 1. Introduction

Post-kala-azar dermal leishmaniasis (PKDL) is a known complication of visceral leishmaniasis (VL) caused by *L. donovani*. It is rare in VL caused by *L. infantum* and *L. chagasi* [1]. In Sudan, PKDL occurs with a frequency of 58% among patients successfully treated for VL, but occasionally it develops in the absence of a pervious history of VL [1–3]. It is considered to be the only reservoir for *L. donovani* in India [4]. In Sudan, VL is both zoonotic and anthroponotic with rodents and canines as candidate reservoirs, at least in some areas endemic for VL [1–3]. Clinically, PKDL lesions are macules, papules, or nodules involving the skin, particularly in the sun-exposed areas [1, 3, 5]. In the majority of Sudanese patients, PKDL occurs within the first two months following treatment of VL [6]. Spontaneous healing occurs in the majority of cases, but in some patients it persists for more than a year [6]. Persistent PKDL is difficult to treat. There is strong evidence that PKDL is immunologically mediated, but the immunological changes involved and their role in the pathogenesis of the disease are not known with certainty.

In the majority of cases, PKDL can be diagnosed on the basis of clinical appearance and distribution of the lesions and a past history of treated VL. We previously described 35 differential diagnoses for PKDL [7]. Of the infectious diseases, leprosy was the most frequent. Some patients do not give a history of VL and these patients are easily misdiagnosed for other skin conditions as will be shown in this paper. PKDL lesions unlike those of cutaneous leishmaniasis are not volcanic in shape, have fixed peri-oral distribution and accompanying hypo/hyperpigmented macules [7]. The ideal diagnostic method is to demonstrate the parasite in smears, by culture or PCR. In some rare atypical cases, the mere identification of parasites in lesions without typing the parasite may not be enough since cutaneous leishmaniasis caused by *L. major* may simulate PKDL, as described in this paper. Using PCR in a previous study, we could demonstrate *L. donovani* DNA in 80.8% and 82.7% aspirates of lymph nodes and lesions of PKDL patients, respectively [8].

The objective of this paper is to describe cases that were misdiagnosed as PKDL. Some were treated with systemic or topical steroids. This led to the exacerbation of the lesions and a high parasite load in the tissues.

## 2. Materials and Methods

A detailed clinical history was obtained from all the patients and a full clinical examination was conducted. Patients were tested for leishmanin skin reactivity (LST) by the intradermal injection of 0.1 mL of a suspension of dead *L. major* promastigotes (5 × 106 *L. major* promastigotes, stock MRHO/IR/75/ER) prepared by Pasteur Institute of Iran [1]. The test was read 48 hours later. An induration of ≥5 mm was regarded as a positive leishmanin test. Hematological investigations included total white cell count, hemoglobin level, platelets count, and erythrocytes sedimentation (ESR). Other tests were urine analysis and liver function tests. The direct agglutination test (DAT) was performed as previously described [1]. A skin biopsy from lesions was fixed in neutral formalin and processed for histopathology. Sections were stained with H&E in all patients and by immunohistochemistry for the cell phenotypes in one [9]. The cells analyzed included CD3+, CD4+, CD8+, and CD 20+. CD4+FoxP3+ regulatory T (T_reg_) in the peripheral blood were also counted by FACs analysis for one patient. All reagents for immunohistochemistry were from Dako (Denmark). The polymerase chain reaction (PCR) was performed on DNA extracted from biopsies of the lesions as described before [8].

## 3. Patients

### 3.1. Patient No. 1

**Figure 1:**
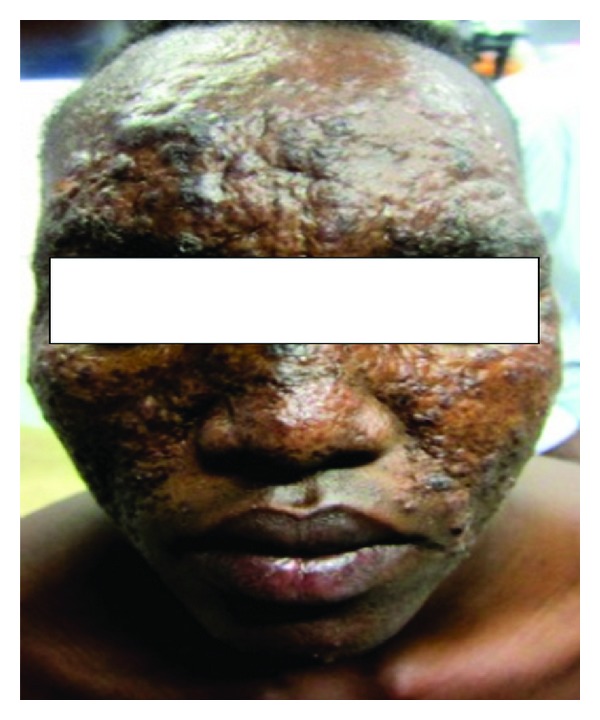
Of patient no. 1 showing papules, nodules, and plaques in the face.

**Figure 2:**
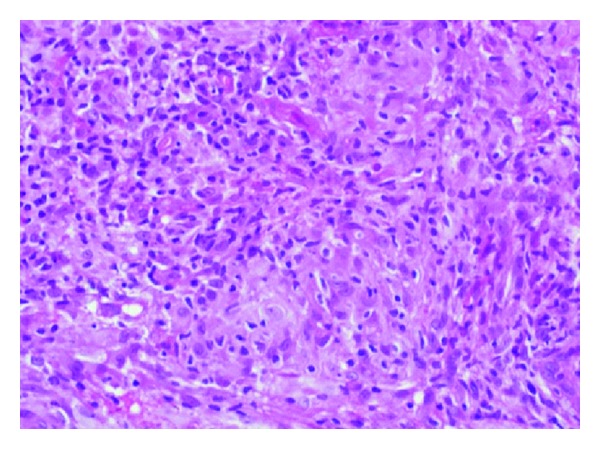
Of patient no. 1 showing histopathological section of lesions showing lymphocytes, macrophages, and poorly formed epithelioid granulomas (H&E stain ×100).

**Figure 3:**
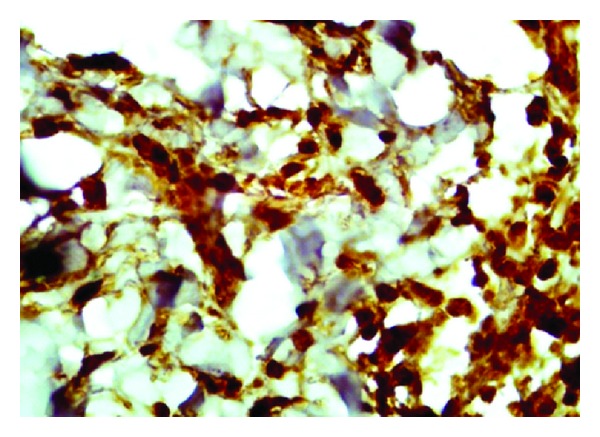
Of patient no. 1 showing leishmania antigen in dermal macrophages. The small round bodies in some cells are probably leishmania parasites that were not detected in the H&E-stained sections.

**Figure 4:**
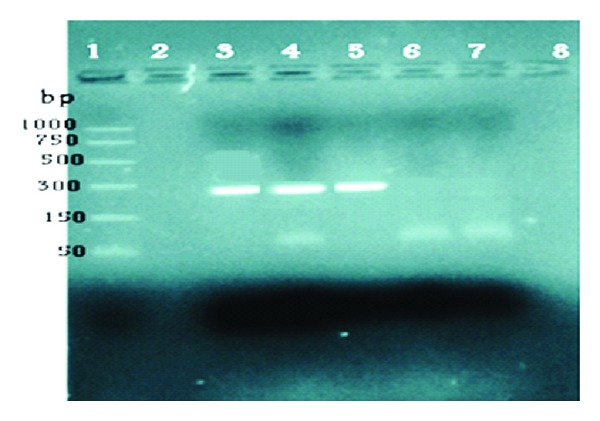
PCR product electrophoresis shows a band measuring 270 base pairs. Line 1: DNA ladder. Line 2: negative control. Line 4: patient no. 3. Lines 3 and 5 are positive controls for *L. major*.

**Figure 5:**
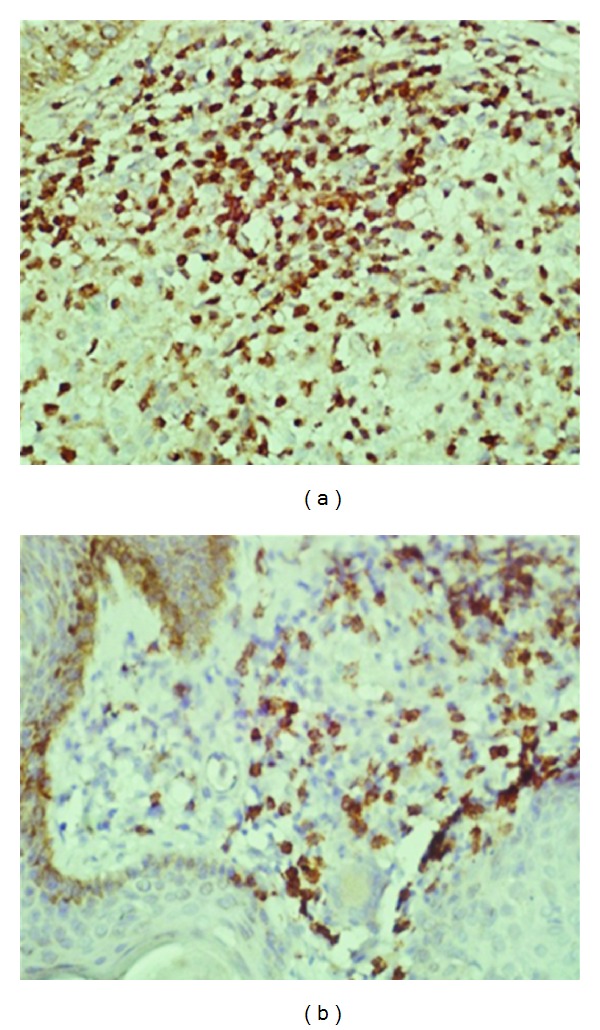
Of patient no. 1. The figures on the left and the right were stained for CD3+ and CD8+ cells. The majority of the cells in (a) are CD3+-positive T cells. (b) shows CD8+-positive cells. The unstained cells are mainly macrophages.

**Figure 6:**
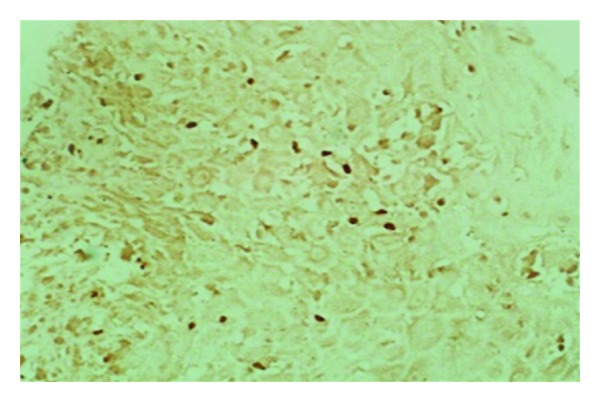
Of patient no. 1. Lesion section shows T_reg_ cells in the inflammatory infiltrate. Normal skin was negative for T_reg_ cells.

**Figure 7:**
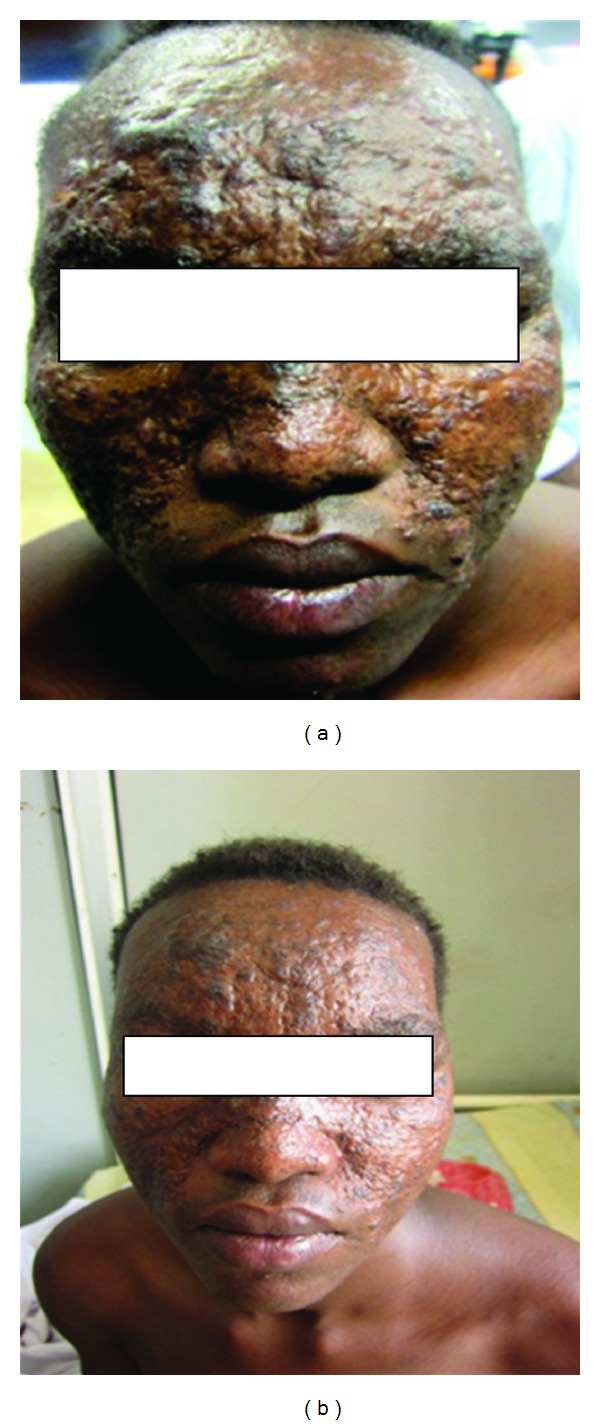
(b) is patient no. 1 before treatment. (a) is the patient just 10 days after treatment with Pentostam. Lesions started to regress and finally healed completely.

This 18-year-old male patient reported with skin rash, mainly in the face of 17 years duration. There was no history of treated VL before the rash. The patient looked well. Liver, spleen, and lymph nodes were not palpable. The rash consisted of nodules and papules (Figure 1). Leishmanin skin test induration was 12 mm. Apart from A/S hemoglobin, there was no significant hematological or biochemical abnormality. A smear was negative for leishmania parasites. A skin biopsy showed lymphocytes, plasma cells, macrophage, and some epithelioid cells (Figure 2). There were few scattered well-formed granulomas. Using indirect immunoperoxidase and primary polyclonal antibody against leishmania prepared in BALBc mice, macrophages and epithelioid cells in the lesions were positive for leishmania antigen (Figure 3). The cells were negative when normal mouse serum and immune serum absorbed with promastigotes were used. All reagents for the immunoperoxidase stain were from Dako (Denmark). PCR showed bands of *L. major* (Figure 4). The majority of the cells were CD3+-positive cells (Figure 5). The majority of the T cells were CD8+-positive cytotoxic cells. CD4+ cells and CD20+ cells were few. In addition, there were T_reg_ cells in the lesions (Figure 6). Using FACs analysis, CD4+FoxP3+ regulatory T (T_reg_) cells in the blood [10] were high: CD4+CD25+ (T_reg_) count was 890 cell/*μ*L compared to in normal Sudanese (~400 cells/*μ*L). The patient was treated with Paromomycin injections at 20 mg/kg/d for 28 days. In ten days, the lesions started to regress (Figure 7) and healed at the end of treatment. 

### 3.2. Patient No. 2

**Figure 8:**
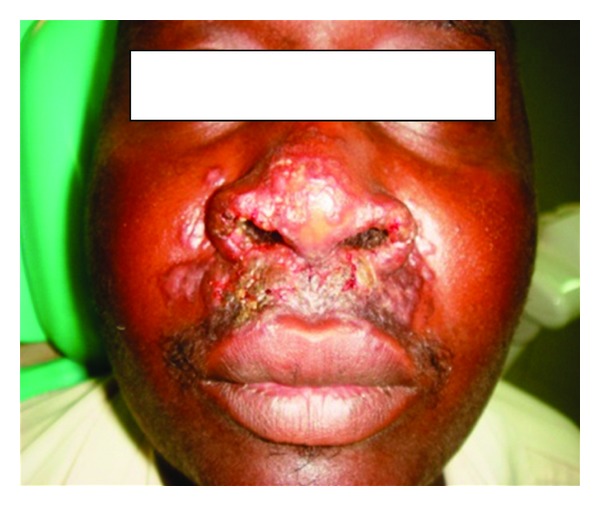
Shows patient no. 2 at presentation. There are papules and plaques over the nose and the lip.

**Figure 9:**
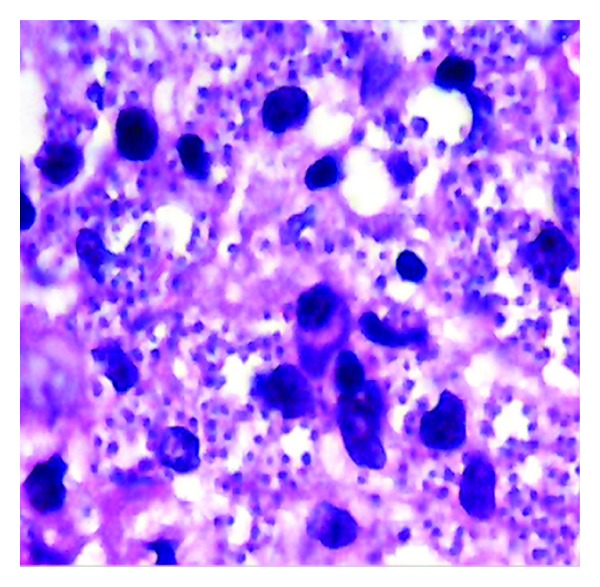
Skin biopsy from patient no. 2 shows many parasitized macrophages. Lymphocytes are scanty.

**Figure 10:**
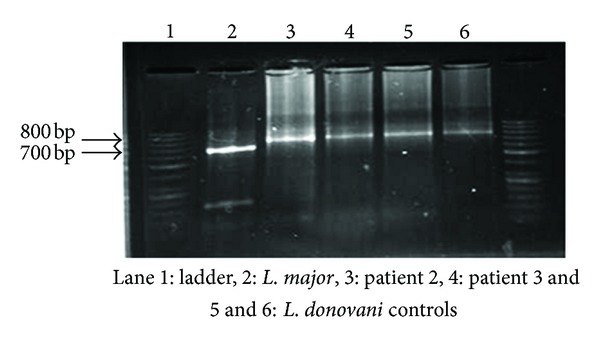
Shows PCR results of patients 2 and 3.

A 35-year-old soldier from Darfur state, western Sudan developed a papular skin rash in the nose of one-year duration with few nodules in the forearms. Before we saw him he was treated elsewhere with topical and systemic steroids, local and systemic antibiotics, and even methotrexate. He mentioned that he became worse. When we saw him, he had a papular rash involving the nose and the upper lip (Figure 8). He gave no previous history of treated VL. His spleen and liver were not palpable. Leishmanin skin test was not reactive. DAT was positive (titre > 6400). A skin biopsy showed heavily parasitized macrophages (Figure 9). Parasites were seen in the epidermis. There were hardly any lymphocytes or plasma cells in the lesion. Haematological and biochemical investigations showed no abnormality. PCR showed the parasite to be *L. donovani* (Figure 10). He was treated with Pentostam at a dose of 20 mg/kg/day for 30 days with full recovery.

### 3.3. Patient No. 3

**Figure 11:**
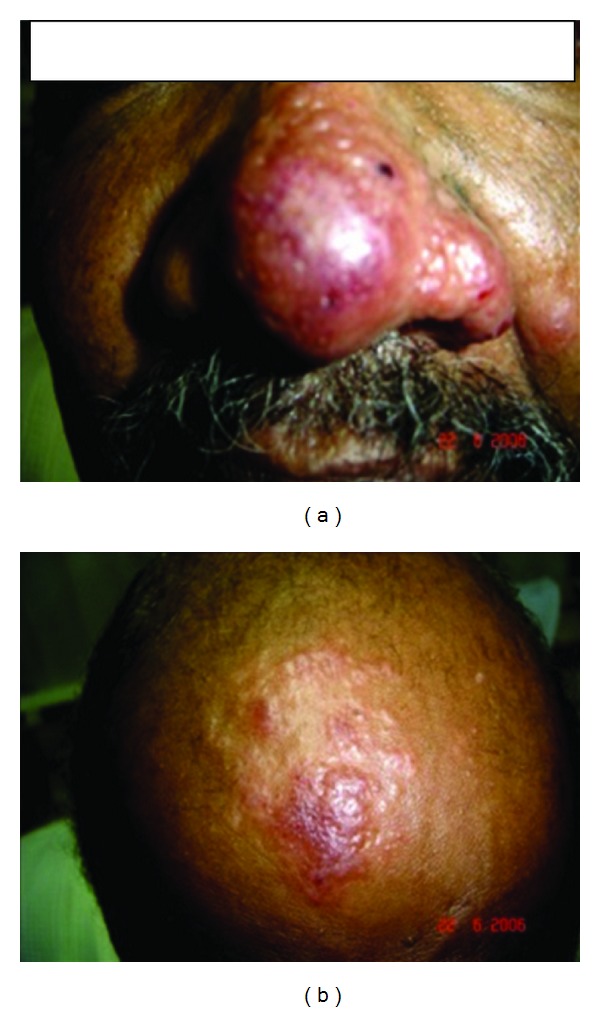
Of patient no. 3 showing lesions in the nose (a) and papules on scalp (b).

**Figure 12:**
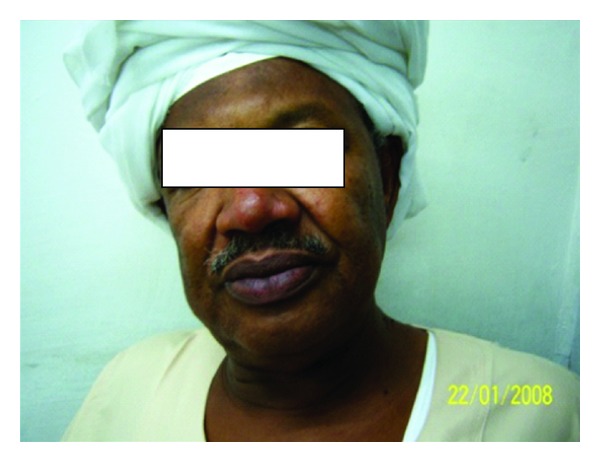
Of patient no. 3 after treatment.

A patient from Gedarif state, eastern Sudan presented to a hospital in Khartoum state with a papular skin rash involving the face. He was diagnosed as lupus pernio and was started on systemic steroids. His lesions became worse. When we saw him, he had papular rash in the scalp and an infiltrative lesion in the nose and lower lip (Figure 11). The inner side of the lip and tongue had a white membrane that proved to be caused by *Candida* infection. The spleen and liver were not enlarged. His leishmanin skin test was not reactive and DAT was positive. A biopsy from the nose showed many macrophages containing leishmania parasites. Lymphocytes were scanty. This is typical of an anergic reaction to leishmania parasites. The parasite was typed as *L. donovani* by PCR. He was treated with AmBisome (2.5 mg/kg body weight/14 days). He completely recovered and remained well at 3 years of followup (Figure 12). 

### 3.4. Patient No. 4

**Figure 13:**
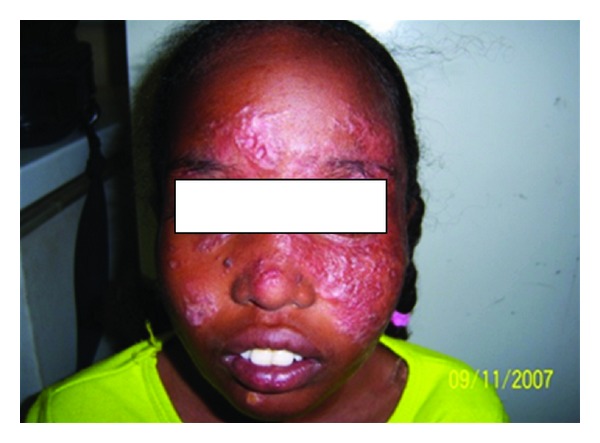
Of patient no. 4 showing papular rash on the face.

This 12-year-old girl was a poorly controlled type I diabetes mellitus. She developed a skin nodule in the forearm. She was treated with topical steroids. She developed disseminated skin lesion in the face, arms, trunk, and legs (Figure 13). Lesions persisted for 7 years. The liver and spleen were enlarged. A biopsy showed leishmania parasites in macrophages inside parasitophorous vacuoles. She was leishmanin-negative and DAT-positive. Her diabetes responded to insulin. After treatment with AmBisome (2.5 mg/kg body weight/14 days) and control of diabetes, the lesions started drying up in two weeks. Smears from the lesions were negative. Three months later, the spleen and liver decreased but were still palpable. She was lost for further followup.

## 4. Discussion

The patients described illustrate some of the conditions that may be mistaken for PKDL. The first patient was referred to us as PKDL. Clinically, he had papules, nodules, and plaques in the exposed parts of the body. The unusual feature was the persistence of lesions for 17 years, since the patient was a year old. Apart from the unsightly appearance, the patient was in good health and developed normally. He was sent to school, but disrupted his education early in his life and he never had a job. Consequently, he was in a state of depression for years. Perhaps the people in his village might have suspected that he had leprosy which is considered a stigma in some parts of Sudan. To our surprise, he turned out to have cutaneous leishmaniasis caused by *L. major* and not PKDL. The part of Darfur where he came from is a known endemic area for cutaneous leishmaniasis. 

Pathologically the patient had a granulomatous inflammatory reaction with a low parasite load. However, there was leishmania antigen in the macrophages and epithelioid granulomas. This requires cytotoxic T cells that are able to recognize the antigen and destroy the cells that exhibit it on their outer membrane. The majority of the cells were CD3+ T cells. Most of the T cells were CD8+ cytotoxic cells. Leishmania parasites were few but were easy to detect by PCR. Under normal circumstances in self-healing cutaneous leishmaniasis, the parasites never disappear completely from the tissues and persist in scars after self- or drug-induced healing [11]. The achievement of latency provides clear benefits to the host. In addition to halting microbe-mediated damage, it was shown that latent infection can be essential for the maintenance of immunity to reinfection [12]. On the other hand, latency allows for the possibility of disease reactivation and facilitates microbial dispersal to new hosts [12]. Latency can, thus, benefit both host and pathogen. Using murine models, it was reported that CD4+CD25+ regulatory T cells (T_reg_) are essential for the development and maintenance of latent cutaneous infection of *L. major* [13]. T_reg_ rapidly accumulate at sites of infection with *L. major*, suppressing the ability of the immune response to eliminate the parasite. There were many T_reg_ cells in the lesions and in the peripheral blood of our patient. T_reg_ cells are known to downregulate the function of CD+8 cells. 

The use of topical and systemic steroids has led to exacerbations of infection in three of the patients described here. Steroids are useful drugs if used when indicated. They are useful adjuncts in treating leishmanial anterior Uveitis [14], leprosy reactions, and spinal schistosomiasis. They are contraindicated in PKDL, cutaneous leishmaniasis, and zygomycosis. An accurate diagnosis must be made before steroids are used.

**Figure 14:**
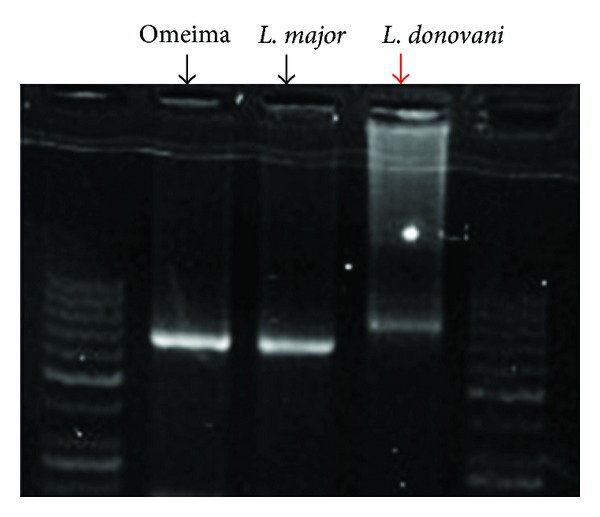
PCR of patient no. 4 (Omeima) showing *L. major* band with *L. major* and *L. donovani* controls.

The diabetic young girl had disseminated *L. major* infection in the skin (Figure 14) and probably in the spleen and liver. Evidence for this was the reduction of the hepatosplenomegaly after antileishmanial therapy. Diabetes mellitus has long been considered an important risk factor for infection and is indeed recognized by the World Health Organization as a cause of secondary immunodeficiency [15]. When the patient was treated with steroids, T-cell-mediated immunity was depressed even more.

## 5. Conclusion

Injudicious use of topical and systemic steroids should be discouraged unless a definitive dermatological diagnosis is made especially in sporadic dermatoses in patients from VL endemic areas but who do not give a history of treated VL. Those patients may have had subclinical *L. donovani* infection. Demonstration of the leishmania antigen/DNA is mandatory in sporadic cases before initiating antimonial or liposomal treatment.
